# Senescent cells and macrophages: key players for regeneration?

**DOI:** 10.1098/rsob.200309

**Published:** 2020-12-23

**Authors:** Sonia S. Elder, Elaine Emmerson

**Affiliations:** The Centre for Regenerative Medicine, Institute for Regeneration and Repair, The University of Edinburgh, 5 Little France Drive, Edinburgh EH16 4UU, UK

**Keywords:** senescence-associated secretory phenotype, macrophage, regeneration, senescence, ageing, repair

## Abstract

Over the last decade, our understanding of the physiological role of senescent cells has drastically evolved, from merely indicators of cellular stress and ageing to having a central role in regeneration and repair. Increasingly, studies have identified senescent cells and the senescence-associated secretory phenotype (SASP) as being critical in the regenerative process following injury; however, the timing and context at which the senescence programme is activated can lead to distinct outcomes. For example, a transient induction of senescent cells followed by rapid clearance at the early stages following injury promotes repair, while the long-term accumulation of senescent cells impairs tissue function and can lead to organ failure. A key role of the SASP is the recruitment of immune cells to the site of injury and the subsequent elimination of senescent cells. Among these cell types are macrophages, which have well-documented regulatory roles in all stages of regeneration and repair. However, while the role of senescent cells and macrophages in this process is starting to be explored, the specific interactions between these cell types and how these are important in the different stages of injury/reparative response still require further investigation. In this review, we consider the current literature regarding the interaction of these cell types, how their cooperation is important for regeneration and repair, and what questions remain to be answered to advance the field.

## 1. Introduction

Tissue repair and regeneration are critical biological processes which occur following injury and are essential for survival. Injury can occur as a result of infection, toxic or mechanical assault, and results in a prominent activation of the immune system and the recruitment of a vast number and type of cells, which infiltrate the damaged area. These consist of natural killer cells, macrophages, neutrophils, B cells, T cells, fibroblasts, epithelial cells and endothelial cells. In a healthy environment such cells work together in a concerted effort to restore tissue function and to limit damage, a process which must be tightly regulated. In many pathological environments, these mechanisms become dysregulated and the recruitment of immune cells can instead initiate, amplify and even sustain tissue injury. This process of healing damaged tissue is known as ‘repair’ and encompasses the two separate processes of regeneration and replacement, where regeneration refers to the process in which new tissue growth restores areas of damaged tissue to their original state while replacement occurs in severely damaged tissue, often in the form of scarring.

While the inflammatory cascade serves to eliminate the noxious stimulus and clear the injured area from dead cells and matrix debris, the healing of injured tissues is dependent on the timely suppression of inflammation, setting the stage for the activation of reparative cells [1]. However, the effectiveness of the reparative response is dependent on the severity and type of injury, the organ affected and species-specific characteristics. While amphibians can regenerate limbs [2] and fish can regenerate myocardium [3], adult mammals fail to regenerate either of these. Furthermore, in adult mammals organs such as the liver retain some regenerative capacity [4], whereas the regeneration of the brain and spinal cord is extremely limited [5]. To add to this complexity, when tissues are exposed to prolonged injury the process of repair can become chronic or dysregulated, leading to pathological processes, including fibrosis or chronic inflammation, which ultimately impact organ function and can result in organ or organism death.

### 1.1. Cellular senescence

**Figure 1.**
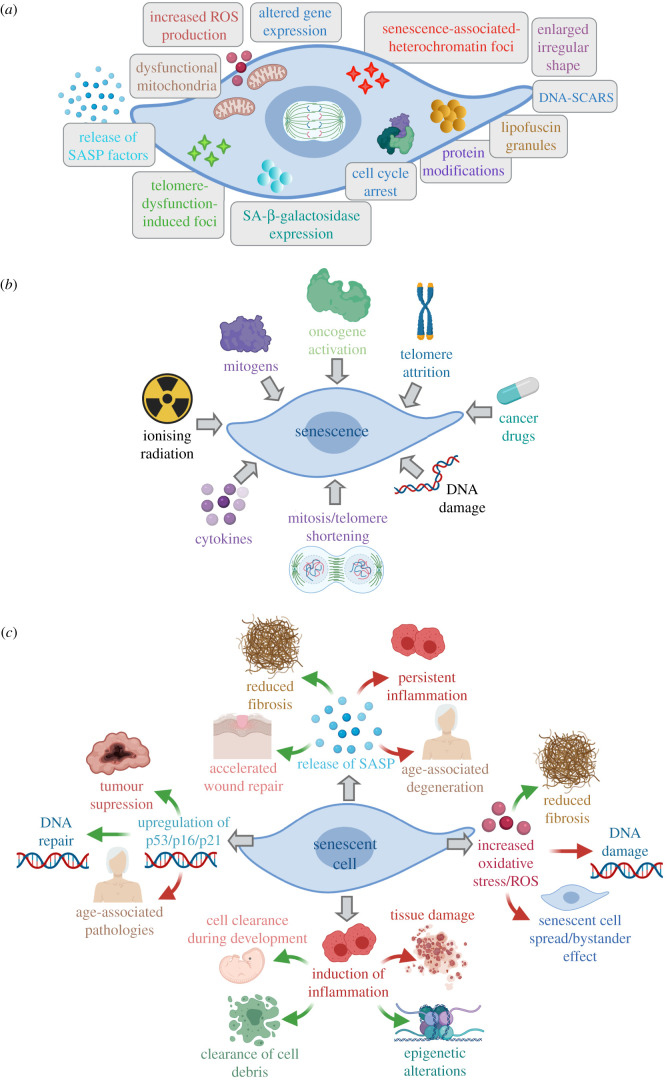
The hallmarks, causes and effects of cellular senescence. (*a*) The key features of a senescent cell, which include an enlarged and irregular/flattened shape [6], DNA segments with chromatin alterations reinforcing senescence (DNA-SCARS) [9] and foci [10], altered gene expression [8] and cell cycle arrest [7], mitochondrial dysfunction and release of reactive oxygen species (ROS) [12,13], protein modifications [14], lipofuscin granules, expression of SA-β-galactosidase [16] and release of SASP factors [14,17]. (*b*) Cellular senescence can occur in response to cellular or DNA damage [18], oncogene, mitogen and cytokine signalling [19], telomere attrition/shortening [20], ionizing radiation [21] and anti-cancer drugs [22]. (*c*) Cellular senescence plays a dual role during development [23,24] and throughout tissue repair and regeneration [25,26], where it can promote the clearance of cell debris, reduces fibrosis, elicits epigenetic alterations and acts as a potent barrier against tumorigenesis, while also leading to senescence spread, DNA damage, further tissue injury, and ultimately leading to age-associated tissue deterioration and pathologies. Created with Biorender.com.

A common outcome of the injury process is cellular senescence, an irreversible but stable form of cell cycle arrest, defined by an altered transcriptome, which occurs in proliferating cells when they have reached the end of their replicative lifespan, or when subjected to stress. Senescent cells are often characterized by an enlarged and flattened shape [6], and exhibit hallmarks of senescence, including DNA and chromatin alterations and gene expression changes [7–11]; mitochondrial dysfunction and the subsequent release of reactive oxygen species (ROS) [12,13]; protein modifications [14] and accumulation of lipofuscin granules [15]; expression of SA-β-galactosidase [16]; and the release of SASP factors [14,17] (figure 1*a*). Moreover, while often found in injured tissues [27,28], senescent cells can also be present in uninjured organs, especially in organs that have previously experienced damage or disease [29], and particularly in older individuals. Cellular senescence was first discovered in primary cell culture, where cells grown for long periods of time, akin to ageing, reached a state where they were no longer able to replicate [30,31]. Subsequently, cells positive for senescence-associated (SA)-β-galactosidase were observed in aged tissues [16]. For many years following this, senescence was solely viewed as a result of organismal ageing; however, in the last decade, our understanding has dramatically evolved, indicating that cellular senescence can occur in response to a range of stimuli, including cellular damage [18], oxidative stress [32], oncogenic signalling [19], telomere attrition [20], ionizing radiation [21] and some cancer drugs [22] (figure 1*b*), and is even seen during development [23,24] (figure 1*c*). Senescence has been reported in numerous cell types during natural ageing, and following injury or disease; including epithelia [33], endothelia [34], immune cells [35], mesenchymal cells [36], bone [37], muscle [38] and adipose tissue [39]. An important role of senescence is therefore to prevent the spread of damage throughout the tissue and, in cancer, acts as a potent barrier against tumorigenesis (reviewed in [40]) (figure 1*c*). In general, the transient induction of senescence followed by senescent cell elimination promotes tissue remodelling and regeneration [25,26] (figure 1*c*); however, chronic injury can result in the long-term accumulation of senescent cells, driving persistent inflammation which ultimately impairs tissue function and can contribute to organ failure (figure 1*c*). For this reason, the fine balance of senescent cells and their presence/clearance is likely to play a pivotal role in tissue repair. In this review, we have concentrated on the literature describing the interplay between senescence in epithelial tissues and immune cells, particularly macrophages.

### 1.2. Senescence-associated secretory phenotype

Senescent cells are often characterized by their ability to develop a senescence-associated secretory phenotype (SASP), a pro-inflammatory response which activates and reinforces the senescence phenotype in surrounding cells, modulates fibrosis and promotes regeneration [41] (figure 1*c*). The SASP consists of a complex mixture of extracellular matrix proteases, growth factors, chemokines and cytokines, which have a profound effect on the tissue microenvironment [42]. Such SASP components can trigger senescence in neighbouring cells in both an autocrine [43,44] and paracrine [41,45] manner, suggesting that senescence creates an inflammatory microenvironment which may lead to the elimination of senescent cells. The secretion of pro-inflammatory cytokines, such as interleukin-6 (IL-6) and interleukin-8 (IL-8) [46], chemokines, such as monocyte chemoattractant proteins (MCPs) and macrophage inflammatory proteins (MIPs) [42], and growth factors, such as transforming growth factor-β (TGF*β*) [47], cause inflammation and recruit immune cells to clear senescent cells.

### 1.3. Macrophages in tissue repair

Among the variety of cell types which orchestrate repair, macrophages have been shown to exhibit critical regulatory activity at all stages of repair and fibrosis. Macrophages are recruited to the site of injury by chemokine gradients and various adhesion molecules, where they carry out their role as scavenger cells that phagocytose cellular debris and invading cells, alongside other apoptotic cells, in response to tissue injury. Importantly, macrophages are key for the clearance of senescent cells following injury [48,49], as well as an important source of chemokines, matrix metalloproteinases (MMPs) and other inflammatory mediators which drive the initial cellular response [50]. Current models suggest that senescence initiates tissue modelling/remodelling by recruiting immune cells through the SASP, where macrophages clear senescent cells, allowing for repopulation by progenitor cells and regeneration of the damaged tissue [25,26,51]. However, in the case of persistent damage or in aged tissues, clearance and regeneration may be compromised due to poor macrophage recruitment, increased senescent cells or even damage to the macrophages themselves. Indeed, if macrophages are depleted in the early stages of repair in a number of organs, the inflammatory response is diminished [52] and leads to less efficient repair and regeneration [53,54].

This review focuses on the recent findings that have advanced our understanding of senescent cells and macrophages in tissue injury, and the importance of the cooperation of these cells as key players in facilitating tissue regeneration and repair.

## 2. Evidence for the role of senescent cells in tissue injury

Senescence is a state of irreversible proliferative arrest, which cells undergo in response to a variety of detrimental stimuli, and is associated with changes in morphology, lysosomal activity, alternations in chromatin structure (H2Ax expression) and activation of the SASP [55] (figure 1*a*). Much of our current understanding of senescence has stemmed from studies of either disease or ageing; however, a novel role for senescence in resolving tissue injury has recently emerged. Indeed, senescent cells have been identified in a variety of injured organs, including the liver [28,47], kidney [56,57], heart [58], skeletal muscle [27] and salivary glands [59], and have largely been associated with the loss of tissue function. Nonetheless, the presence of senescent cells has been reported to have both positive and negative effects in their resident organ, depending on their abundance and duration (figure 1*c*), and provides us with important insights into their physiological function. Indeed, if the function of senescence is the elimination of cells, why do cells not undergo the faster and more direct route of apoptosis, and why were senescent cells selected during evolution? This question has led to the emerging concept that senescent cells play important roles in tissue repair and remodelling, providing a final function before eventually undergoing elimination themselves [26].

### 2.1. The role of senescent cells in tissue repair

To date, two main approaches have been used to explore the role of senescent cells in repair: genetic depletion strategies, where senescent cells are deleted from the tissue, and through the use of senolytics, where compounds are used to induce senescent cell death.

### 2.2. Genetic depletion strategies

**Figure 2.**
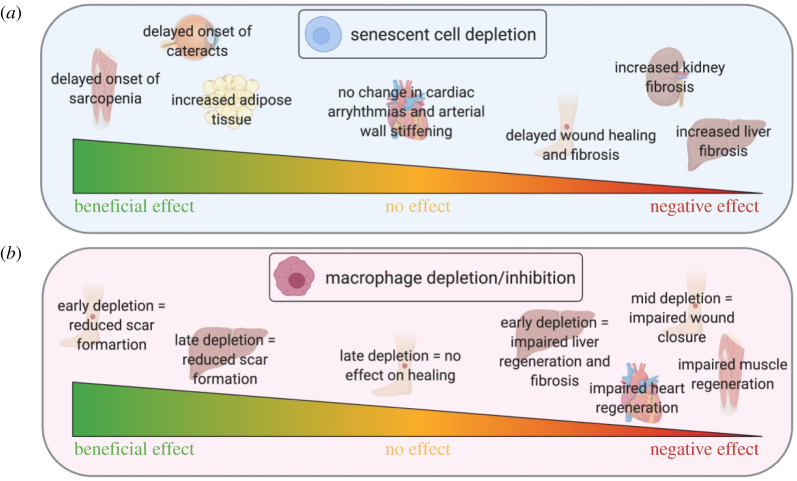
Depletion of macrophages or senescent cells has diverse and opposing effects on organ regeneration. The effects of senescent cell depletion (*a*) or macrophage depletion or prevention of accumulation (*b*) are depicted. In many organs, the timing of cell depletion has a crucial role on the regenerative outcome. While senescent cell depletion delays cutaneous wound healing and exacerbates fibrosis [60,61], the effect of deletion of macrophages during wound healing is timing-dependent [54]; similarly while senescent cell depletion leads to liver fibrosis [28,62], depletion of macrophages can lead to both reduced liver scarring and fibrosis, dependent on timing [52]. Moreover, senescent cell removal has no or largely positive effects on muscle and heart [63]; however, macrophage depletion leads to detrimental effects on heart and muscle regeneration [63–65]. Created with Biorender.com.

To distinguish the role of senescent versus apoptotic cells in tissue injury, Baker *et al.* [66] used an inducible ‘senescence-to-apoptosis' progeric mouse model, where transgenic mice express pro-apoptotic proteins under the expression of the p16^INK4a^ promoter. By administering the mice with a ‘chemical switch’, cells expressing the senescence-associated marker p16^INK4a^ were converted into apoptotic cells *in vivo*. Interestingly, as well as observing a decrease in the number of senescent cells, Baker *et al*. [66] observed a reversion in a number of age-associated pathologies (figure 2), indicating a role for senescent cells in disrupting tissue homeostasis. However, using a similar but mechanistically different mouse model in cutaneous wound healing studies, it was shown by Demaria *et al*. [60] that inducing mice to switch from senescence to apoptosis significantly delayed wound healing and caused the wounds to accumulate larger amounts of fibrotic tissue (figure 2). Interestingly, in young mice, a transient burst of P16^INK4a^+ senescent cells was found to occur during normal wound healing and disappeared following wound closure, indicating an early role for senescent cells in wound recovery and the negative impact of their removal [60]. Similarly, cellular senescence occurs in myofibroblasts in cutaneous wounds during the healing process, which is thought to minimize the extent of fibrosis [61].

In support of these observations, contrasting effects of senescent cell function have also been demonstrated in other models of tissue injury. When the liver is injured, hepatic stellate cells become senescent and produce a stable fibrotic scar [28] (figure 2). *In vivo*, these senescent cells are identified inside the fibrotic lesions; however, mice deficient for *p53* and *p16^INK4A^* show increased fibrosis in both the liver and kidney [28,62]. Conversely, in a mouse model of oncogenic NRAS^G12 V^, where senescent cells are usually cleared by monocytes and macrophages, immunodeficient mice show reduced clearance, resulting in mature liver tumours [67]. Moreover, the p16-3MR transgenic mouse, a model that contains a p16^INK4a^ promoter which allows the tracing and removal of senescent cells has been used to demonstrate that senescent cell deletion reduces pain in an experimental model of osteoarthritis [68]. Crucially, a mouse model that contains the transgene, INK-ATTAC, which induces apoptosis in p16^INK4a^-expressing cells, established that senescent cell clearance treatment extended lifespan in both male and female mice, delayed tumorigenesis and attenuated age-related deterioration of several organs, including kidney, heart and fat, without apparent side effects [69].

### 2.3. Senolytics

The use of senolytic compounds also provides a mechanism to elucidate the role of senescent cells and in particular, the specific timing of depletion. Evidence shows that senolytics can drive the expression of SA-β-gal in cell culture [70]. Moreover, the administration of the senolytic compounds ABT-737 or Dasatinib plus Quercetin (DQ) *in vivo* induces apoptosis in senescent cells and leads to clearance in mouse skin, lung and the haematopoietic system, and subsequently improves tissue repair [70–73]. Moreover, DQ administration promotes the survival of transplants from aged mice [74].

Taken together, these studies highlight the opposing roles of senescent cells in injury and repair, and the variation in their function as a result of timing, degree and type of injury. Indeed, increasing evidence suggests that cellular senescence is a multi-step, dynamic process, progressing from a transient to a stable state of cell cycle arrest, dictating the outcome [75].

### 2.4. The role of the senescence-associated secretory phenotype in tissue repair

Furthermore, beyond the direct effect on cell division and clearance, a key mechanism in which senescent cells influence injury is through the SASP. The SASP secretome includes a wide variety of soluble signals capable of influencing tissue inflammation, repair and fibrosis, including IL-1, IL-8, IL-6 and transforming growth factor beta (TGF*β*). The SASP is a pro-inflammatory response which activates and reinforces the senescent phenotype in surrounding cells and therefore mediates the spread of senescence throughout the tissue, known as ‘bystander senescence’. Through a variable set of cytokines and chemokines, paracrine senescence is induced and maintained through mechanisms which generate reactive oxygen species (ROS) and the DNA-damage response (DDR) [76]. For example, paracrine senescence has been shown to exacerbate biliary injury and impair regeneration in the liver [47]. Upon the partial ablation of the SASP, through TGF*β* inhibition, hepatocyte proliferation is increased, fibrosis is decreased, and overall liver function is improved. Opposingly, in the mouse model of cutaneous skin injury employed by Demaria *et al.*, the authors identified a positive effect of the SASP [60]. Intriguingly, through the secretion of the SASP factor PDGF-AA, the closure of skin wounds was found to be accelerated, by promoting myofibroblast differentiation and granulation tissue formation.

Indeed, when the SASP was first studied, its role in injury and resolution was not well understood, as the components appeared to be mostly pro-inflammatory. It has since been suggested that senescence may impair regeneration through paracrine signalling, leaving neighbouring cells unable to compensate for damage, resulting in enhanced fibrosis and a diminished capacity of the regenerative response [47]. However, it appears that the prominent inflammatory components and detrimental consequences occur largely when the SASP is long-lived and may be beneficial when transient, thus playing a critical role in tissue remodelling at the early stages of injury. In fact, in 2017, Chiche *et al*. [29] showed that in acute and chronic injury, the release of the SASP factor IL-6 from senescent cells enabled the reprogramming of muscle satellite cells, indicating a role for the SASP in facilitating cellular plasticity and repair. Importantly, this points to a beneficial role of the SASP in promoting the cellular plasticity of stem cell populations during acute muscle injury, as well as in the pathological setting of muscle deterioration [29].

### 2.5. Cellular senescence in pluripotency

To add to the aforementioned findings, a recent study has demonstrated that the reprogramming factors OCT4, SOX2, KLF4 and cMYC can induce cellular senescence and IL-6 production *in vivo*, which leads to more efficient reprogramming [77]. Moreover, SASP can promote a pro-regenerative response through the induction of plasticity and stemness in somatic stem/progenitor cells [78]. Ritschka *et al*. [78] showed that a transient exposure to the SASP in primary mouse keratinocytes caused the increased expression of stem cell markers and regenerative capacity *in vivo*. However, prolonged exposure to the SASP triggered senescence arrest which countered the regenerative stimuli [78]. It has thus been proposed that in the case of injury, the senescent cell uses the SASP to induce plasticity and stemness in neighbouring cells, enabling the replacement of the senescent cell once it has been cleared and encouraging tissue regeneration [25]. However, it is important to note, when this process is uncontrolled, senescence-associated reprogramming can lead to tumour formation by promoting cancer stemness [79]. By contrast, SASP factors which are secreted by senescent cardiac progenitor cells (CPCs) via paracrine signalling result in the senescence of otherwise healthy CPCs. In this context, the elimination of senescent cells in aged mice or in mice treated with senolytics abrogated the SASP and resulted in the activation of resident CPCs and an increase in the number of proliferating Ki-67 EdU+ cardiomyocytes, thus indicating that the removal of senescent cells may alleviate deterioration following cardiac injury and contribute to the capacity of the heart to regenerate [58]. Moreover, the deletion of the senescence effectors p53 and p16^INK4a^ improves the reprogramming efficiency of human fibroblasts to iPSCs, suggesting, that in this environment, senescence has a negative effect on plasticity [80]. Importantly, these differences in whether senescent cells are beneficial/detrimental to regeneration highlight the importance of the timing in which the senescence program is activated and its changing role during different stages of the injury response.

To date the SASP is known to have important roles in embryonic development, wound healing and tumour growth, indicating that the SASP has more complex physiological roles than we currently understand (reviewed in [81,82]). Taken together these studies demonstrate that senescent cells play important roles in tissue injury and regeneration and can both promote and inhibit tissue repair. Simply, the evidence of the positive effects of senescent cell removal comes from the circumstances where senescent cells accumulate and lead to negative consequences. Conversely, a transient wave of senescent cells appears to play an important role in promoting repair in the early stages of injury. Overall, these findings support the understanding that the senescence programme can be a beneficial regenerative process; however, when it is perturbed, it can play a detrimental role. For example, while acute senescence clearly plays an important role in preventing malignancy and promoting successful tissue repair, the accumulation of chronically senescent cells further contributes to injury, disease and ageing.

## 3. Role of macrophages in tissue injury

**Figure 3.**
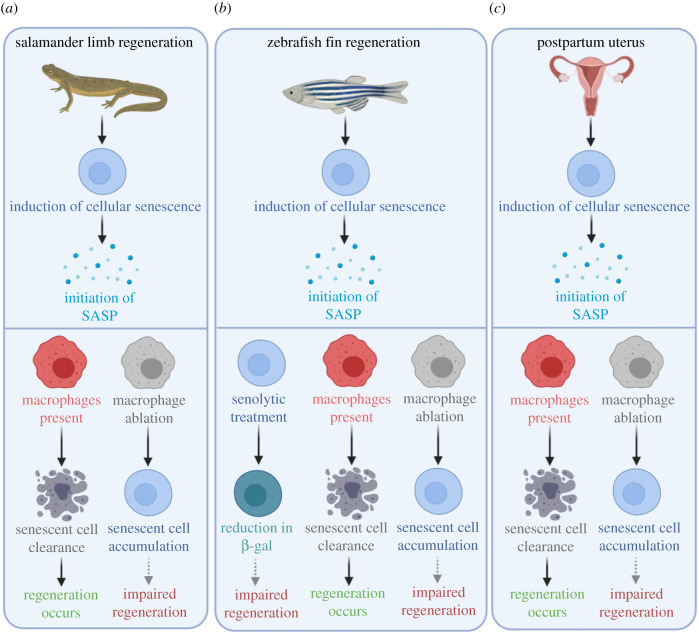
Animal models of regeneration have provided evidence of the interplay between macrophages and senescent cells during tissue regeneration. The induction of cellular senescence leads to the initiation of the senescence-associated secretory phenotype (SASP) during salamander limb regeneration, zebrafish fin regeneration and post-partum uterus regeneration. In the absence of macrophages, senescent cells in the regenerating salamander limb are not cleared, which is a possible reason for impaired regeneration (dashed grey arrow) [48]. The removal of either senescent cells or macrophages during zebrafish regeneration has a deleterious effect on regeneration, hypothesized to be as a result of altering the tightly regulated balance of cell senescence (dashed grey arrows) [125]. The mammalian uterus undergoes extensive remodelling post-partum where senescent cells are normally cleared by macrophages. In the absence of macrophages, senescent cells accumulate in the uterus [126], presumably leading to dysregulated regeneration and function (dashed grey arrow). Created with Biorender.com.

While a large variety of cell types have been shown to play important roles in injury and repair, in recent years, a particular interest in macrophages has developed, due to the contribution of different macrophage populations and their plasticity in the context of injury. Thus, in recent years, there has been a particular focus on the identification of different macrophage states and subsets in many different organ systems and their different roles in injury and repair. Macrophages are crucial for limb regeneration in the salamander [83] and tail fin regeneration in the zebrafish [84] (figure 3). Moreover, macrophages interact intimately with their surroundings integrating cues from invading pathogens, commensal bacteria, as well as tissue-specific functions, rendering macrophages extremely well adapted to their local environment and thus acquiring organ-specific functions [85,86]. The macrophage populations which are found in the many different tissues of the body are termed ‘tissue-resident’ macrophages and are largely derived from the yolk-sac during embryogenesis (reviewed in [87]). As long-lived cells, tissue-resident macrophages are particularly important, due to their witnessing and ‘memorizing’ past and present events in the tissue, which plays an important role in their plasticity. Furthermore, it has been shown that these macrophages are capable of readjusting their behaviours, probably through epigenetic modifications [88,89]. Therefore, tissue-resident macrophages are critical in maintaining tissue homeostasis.

In response to tissue injury, inflammation results in an initial influx of neutrophils, accompanied by monocyte-derived macrophages, which clear cellular debris and coordinate cellular processes to initiate tissue repair. Importantly, this process leads to an overall diluting of the tissue-resident macrophages in the macrophage pool, which is further exacerbated by the proliferation of infiltrating macrophages, which adapt their function to surrounding cues in the local microenvironment. A range of studies have thus identified the specialized roles of monocytes and macrophages and the timing of their activation as critical in the various steps of tissue repair, regeneration and remodelling [52,54].

### 3.1. Macrophage phenotypes

In the past, macrophages have been broadly separated into two categories: M1 (classically activated) and M2 (alternatively activated), based on their inflammatory and anti-inflammatory/reparative functions, respectively. Nowadays, the binary M1/M2 classification is generally considered an oversimplification of the large variety of macrophage populations that exist *in vivo*, and to date have been further subdivided, based on their gene expression profiles [90,91]. Nonetheless, pro-inflammatory macrophages are generally associated with the expression of high levels of pro-inflammatory cytokines, an ability to mediate resistance to pathogens, produce reactive nitrogen and oxygen intermediates, and promote *Th1* responses [92]. On the other hand, reparative macrophages are characterized by their role in tissue remodelling and repair, regulation of the immune system, scavenging and phagocytic capabilities [93], thus, exerting mainly pro-tumoral and immunoregulatory functions (reviewed in [94]).

Unsurprisingly, the presence of pro-inflammatory macrophages has been shown to sustain tissue-damaging inflammatory responses, and the presence of these cells has been associated with a variety of inflammatory and fibrotic diseases. The role of pro-inflammatory macrophages has been particularly well-studied in models of spinal cord injury, where macrophages have been shown to readily accumulate at the site of injury. In these models, macrophage activation and polarization, depending on changes in the microenvironment, has shown that the sustained recruitment of pro-inflammatory macrophages facilitates axonal dieback and can substantially delay the regenerative response [95], and their death *in situ* further contributes to tissue damage [96]. Furthermore, the presence of axon growth inhibitors is significantly higher in pro-versus anti-inflammatory macrophages, suggesting that these cells can actively contribute to suppressing regeneration after spinal cord injury [97]. In addition, studies in the liver have also implicated inflammatory macrophages in exacerbating injury, where an increase in inflammatory macrophages is observed in areas of hepatic necrosis [98,99]. This has also been observed during acute kidney injury where inhibition of early, pro-inflammatory macrophages improves renal function [100] (figure 2). However, it is important to note that pro-inflammatory macrophages may also contribute to the processes which lead to recovery. This has been observed in models of skeletal muscle injury, where inhibition of monocyte/macrophage accumulation impairs muscle regeneration [63,64], and cardiac regeneration, where macrophage depletion leads to alterations in myofibroblast infiltration and neovascularization, and subsequent ventricular dilatation and mortality [65] (figure 2). Thus, a fine balance between pro/anti-inflammatory macrophages is probably needed for optimal repair following injury.

### 3.2. Macrophage depletion and reconstitution studies

Perhaps unsurprisingly, the complete depletion of macrophages has also been found detrimental for tissue repair. Depletion studies in models of tissue injury in the liver have shown that macrophage-depleted mice fail to exert a complete cytokine response, which subsequently compromised liver regeneration [52]. This is likely to be due to the loss of anti-inflammatory pro-regenerative macrophages which play critical roles in promoting tissue repair. These macrophage populations have been partially defined by their production of the anti-inflammatory cytokine IL-10, which functions as an important anti-inflammatory mediator essential for the maintenance of anti-inflammatory activity [101]. Interestingly, in a model of early-onset inflammatory bowel disease, the loss of the IL-10 receptor (IL-10R) resulted in the spontaneous development of colitis [102], indicating that IL-10R signalling in intestinal macrophages is an important factor for controlling intestinal inflammation. Moreover, while cutaneous wound healing is accelerated in mice deficient for IL-10, a result attributed to accelerated re-epithelialization and wound contraction, macrophage infiltration was significantly elevated [103], further implicating IL-10 signalling in inflammation.

Furthermore, a recent paper by Podaru *et al.* [104] showed that the transplantation of functional ‘reparative macrophages', acquired from bone marrow mononuclear cells, in a mouse myocardial infarction model, resulted in significant improvement in functional recovery. Here, the authors found that transplantation of the reparative macrophages enhanced myocardial tissue repair, by promoting the formation of the vasculature and reducing cardiomyocyte hypertrophy and interstitial fibrosis. Interestingly, the transplantation of such reparative macrophages was also found to increase the number of their host-derived counterparts, which was partly mediated by TGF*β* secretion [104]. Moreover, following hepatocyte death during liver injury, the engulfment of debris by macrophages leads to the induction of *Wnt3a*, which subsequently leads to canonical WNT signalling in nearby hepatic progenitor cells, facilitating their differentiation into hepatocytes and therefore contributing towards the regenerative response [105]. Thus, inflammatory cell-mediated cytokine signalling plays an integral role in regeneration and tissue resolution.

It has recently been shown that small extracellular vesicles (sEVs) derived from M2 bone marrow-derived monocytes (BMDMs) can attenuate spinal cord injury (SCI). sEVs mediate paracrine signalling and are important for regulating cellular function [106]. Here, sEVs from M2 BMDMs were found to protect neurons in SCI mice, by inhibiting the mTOR pathway and enhancing the autophagy ability of neurons, therefore reducing apoptosis *in vitro* and *in vivo*. This was found to be due to the transfer of the microRNA miR-421-3p, which regulates the mTOR pathway inside the M2 BMDM-sEVs [107]. This study showed for the first time that M2-derived BMDMs are important in protecting neurons and facilitating recovery following SCI, as well as highlighting that this occurs via the transfer of sEVs. Furthermore, this study thus further elucidates the beneficial roles for M2 macrophages during injury and indicates a mechanism by which they carry out their protective/reparative function.

Interestingly, such anti-inflammatory macrophages have been shown to not only promote tissue repair but also antagonize the function of pro-inflammatory macrophages and fight against their pro-fibrotic capabilities [108,109]. Thus, the interaction/cooperation of macrophages with various cell types, including those involved in the initial phase of inflammation, is of vital importance. It has recently been shown that neutrophils also have a crucial function in liver repair, by promoting the phenotypic conversion of pro-inflammatory Ly6C^hi^CX3CR1^lo^ monocytes/macrophages to pro-reparative Lyc6^lo^CX3CR1^hi^ macrophages. Furthermore, this conversion was found to be dependent on the expression of reactive oxygen species (ROS) from neutrophils [110,111]. Intriguingly, this study demonstrated the cooperation between neutrophils and macrophages and the importance of their interaction in the resolution of inflammation and tissue repair [111]. This supports the accumulating evidence that monocyte-derived macrophages can undergo both phenotypic and functional transition in order to promote tissue regeneration and healing [112,113].

Finally, macrophage activity during different phases of tissue injury is important for tissue repair. By selectively depleting macrophage populations Duffield *et al*. [52] showed that macrophages have distinct, opposing roles during injury and repair. Specifically, in a mouse model of Ccl4-induced reversible liver injury, the depletion of macrophages during advanced fibrosis resulted in reduced scarring. However, if macrophages were depleted during the repair period this resulted in the failure of matrix degradation and a persistent activation of the fibrotic response. Importantly, this showed that macrophages perform both injury-inducing and repair-promoting tasks (figure 2), and that functionally distinct subpopulations of macrophages exist within the same tissue that play important roles in different phases of injury/recovery [52]. Moreover, the depletion of macrophages in the early stages of cutaneous wound repair delayed re-epithelialization, leading to reduced scar formation, while depletion in the mid-phase of new tissue formation led to an impaired wound closure. Crucially, depletion in the late stages of repair had no effect on the overall repair response, suggesting that macrophages play different and distinct functions during the phases of skin repair [54,114] (figure 2). Ultimately, studies such as these demonstrate that macrophages exert different and distinct functions at different stages of the repair or regeneration processes; a discovery that is crucial if we are to therapeutically manipulate macrophage function in the future to improve organ regeneration.

## 4. The interaction between senescent cells and macrophages in tissue regeneration and repair

Senescence is now known to play important roles in many different tissues during murine embryonic development [23,24]. This includes in the mesonephric tubules during mesonephros involution (development of the kidneys and testes), the endolymphatic sac of the inner ear, the apical ectodermal ridge of the limbs, the regressing interdigital webs of the hands and feet, and the closing of the neural tube [23]. Indeed, there is evidence that senescent cells in murine development are surrounded by macrophages at days E13.5–14.5, and that the infiltration of macrophages leads to senescent cell clearance and the promotion of tissue remodelling [23,97]. Importantly, the processes that are observed during development provide us with a unique insight into the mechanisms which drive regeneration and repair following injury in adulthood, and act as a starting point for the manipulation of such processes *in vivo*.

### 4.1. Senescent cell surveillance by macrophages

The role of macrophages in clearing senescent cells has been known for more than a decade [115]. Due to the substantial release of cytokines and chemokines by senescent cells via the SASP, it is unsurprising that an increasing number of studies appear to support a macrophage-dependent surveillance mechanism which operates in both normal and regenerating tissues. Indeed, it seems logical that the number of senescent cells must be closely monitored to maintain tissue homeostasis and to mitigate and/or prevent the negative impacts of senescent cell accumulation. The first evidence of the involvement of the immune system in the surveillance of senescent cells came in 2007 from Xue *et al.* [116], who revealed that the reactivation of p53 in p53-deficient tumours led to complete tumour regression. This was found to be mediated by the upregulation of inflammatory cytokines and the activation of the innate immune response [116]. After this, the role of immune cells, including macrophages, were shown to be important in the removal of senescent cells in models of liver injury, as well as for preventing excessive detrimental fibrosis and in resolving liver fibrosis [117]. Moreover, senescent hepatic stellate cells have been shown to secrete a SASP that attracts macrophages [118]. In 2013, Lujambio *et al.* showed that p53-expressing stellate cells release IFN-γ and IL-6, which promote resident Kupffer macrophages and infiltrated macrophages polarize towards a tumour-inhibiting M1 state, capable of targeting senescent cells in culture. However, in senescent stellate cells lacking p53, IL-4 was produced, causing macrophage polarization towards the pro-survival M2 phenotype [119]. Thus, this evidence suggests that senescent cells can elicit phenotypic changes in macrophages which can affect their functionality. Interestingly, mesenchymal stem cells (MSCs) also appear to play a regulatory role in shifting local macrophages from a pro-inflammatory to a tissue reparative phenotype [120,121], and in the absence of macrophages, neonates lose their ability to regenerate their myocardia following myocardial infarction [122].

To add to this, senescent cells are also capable of eliciting an adaptive immune response where they are cleared by CD4+ T cells and monocytes/macrophages. This has been shown in the liver, where pre-malignant senescent hepatocytes undergo clearance by CD4+ T cells, which require the presence of monocytes/macrophages, highlighting the importance of immune cell cross-talk in senescent cell clearance [123]. Furthermore, in a model of liver cancer, senescent cell surveillance was shown to require the recruitment and maturation of CCR2+ myeloid cells, while the ablation of CCR2 caused outgrowth of hepatocellular carcinomas [124].

### 4.2. Senescent cells and macrophage interactions during regeneration

To explore these interactions further, in 2015, Yun *et al.* [48] showed that macrophages were critical for the clearance of senescent cells, providing the first evidence that senescence-surveillance mechanisms operate during normal regeneration. Here the authors demonstrated that during salamander limb regeneration, there was a significant induction of cellular senescence, indicating its importance as a mechanism for regeneration in normal regenerating tissues. Furthermore, Yun *et al*. also reported that a strong SASP signature in blastemas coincided with a strong peak in the induction of senescent cells (figure 3), indicating that these could have paracrine effects on regeneration and the chemo-attraction of macrophages [48]. Crucially, macrophages and senescent cells are found in close proximity to each other in regenerating limbs. By contrast, clodronate-mediated deletion of macrophages results in the persistence of senescent cells during limb regeneration [48]. In support of this, reports in other model organisms such as the zebrafish have demonstrated the negative impacts of macrophage ablation during zebrafish fin regeneration [84]. This has been further investigated in a 2020 study by Da Silva-Álvarez *et al.*, who showed that following injury in the zebrafish, senescent cells were present at the site of injury, and that their removal impaired regeneration [125] (figure 3). Moreover, senescent cells that accumulate in the post-partum mammalian uterus are efficiently cleared by macrophages after birth, while macrophage depletion leads to abnormal accumulation of senescent cells [126] (figure 2).

**Figure 4.**
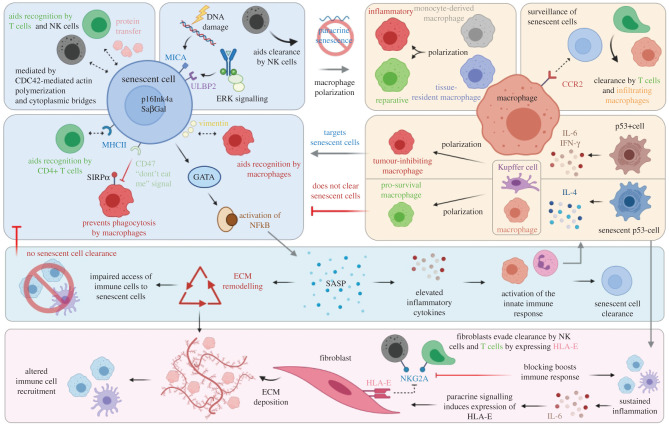
Senescent cells and macrophages communicate via complex bi-directional signals during tissue remodelling, repair, regeneration and tumorigenesis. Senescent cells are detected through immune surveillance by natural killer (NK) cells, mediated by cell–cell contact, protein transfer, actin polymerization and cytoplasmic bridges, which ultimately aids recognition by NK cells and T cells [130]. Senescent cells express the ligands MICA and ULBP2 on their cell surface in response to DNA damage and ERK signalling, which aids in their clearance by NK cells [131]. In addition, senescent cells often express MHCII and vimentin which allows recognition by T cells and macrophages, respectively [123,132,133]. Conversely, senescent cells can evade detection by expressing the ‘don't eat me’ signal CD47, which impairs phagocytosis by macrophages by binding to the inhibitory receptor SIRP*α* [111] and GATA4 is stabilized during cellular senescence, which in turn activates NF*κ*B to facilitate SASP [129]. Macrophages exist as tissue-resident cells or are derived from blood-borne monocytes and can be polarized towards a pro-inflammatory or pro-reparative state. Expression of p16^Ink4a^ and Sa*β*Gal in macrophages has been implicated in a role for polarization, rather than as a consequence of paracrine senescence [128,134,135]. CCR2 expressed by macrophages is critical for senescent cell surveillance and clearance by T cells and infiltrating macrophages [124]. Under normal physiological conditions, p53+ stellate cells release the pro-inflammatory cytokines, IFN-γ and IL-6, which promote resident Kupffer cells and infiltrating macrophages to polarize towards a pro-inflammatory state, which can target senescent cells. Conversely, in senescent stellate cells lacking p53, IL-4 is produced, resulting in macrophage polarization towards the pro-survival phenotype [119]. Senescence-associated secretory phenotype (SASP) leads to elevated inflammatory cytokines, activation of the immune response and senescent cell clearance. In addition to this classical role, SASP also leads to extracellular remodelling (ECM) which contributes to altered cell recruitment and impaired access of immune cells to senescent cells [136]. The MHC molecule HLA-E, which is expressed by senescent cells and induced by IL-6, interacts with the inhibitory receptor NKG2A, expressed by NK cells and T cells, to evade surveillance, and blocking this interaction improves the immune responses against senescent cells [137], in a manner whereby sustained inflammation also contributes to the persistence of senescent cells. Created with Biorender.com.

There therefore exists convincing evidence for the role of senescent cells and their interaction with macrophages in tissue injury and repair. SASP promotes macrophage proliferation [127], while P16INK4a+ macrophages accumulate with increasing age and exacerbate SASP [128]. The transcription factor GATA4 is stabilized during cellular senescence, which in turn activates NF*κ*B to facilitate SASP [129] (figure 4). However, to date, questions remain regarding the mechanisms by which senescent cells interact with the immune system, including macrophages, and how this elicits or prevents a reparative response. It has previously been shown that senescent cells express NKG2D ligands MICA and ULBP2 on their cell surface, allowing their recognition and elimination by natural killer (NK) cells. Furthermore, the expression of these ligands has been found to be regulated by the DNA damage response and the ERK signalling pathway as a result of injury [131] (figure 4).

### 4.3. Senescent cells and macrophage interactions with ageing

Another outstanding question is what allows senescent cells to accumulate during ageing/injury and how senescent cells manage to escape being cleared by the immune system. Progress in answering this question has shown that senescent dermal fibroblasts can evade clearance by the immune system by expressing the non-classical MHC molecule HLA-E. HLA-E functions by interacting with inhibitory NKG2 receptors, expressed by NK cells and CD8+ T cells, to inhibit immune responses against senescent cells (figure 4). The authors found that blocking the interaction between HLA-E and the receptor NKG2A boosted immune responses against senescent cells *in vitro*. Interestingly, they found that the SASP-related cytokine IL-6 induced the expression of HLA-E in non-senescent cells in a paracrine fashion [137]. The upregulation of HLA-E expression by IL-6 thus suggests that sustained inflammation may also contribute to the persistence of senescent cells in tissue, further contributing to the pathogenesis of injury or age-related diseases. This is supported by the fact that IL-6 has been well characterized in senescence [138] and is found in the serum of elderly patients, as well as in various injury models [139,140]. Furthermore, as the authors note, the expansion of CD8+ T cells which are NKG2C+, another isoform of the inhibitory NKG2 receptors, with age may offer an explanation as to why the immune system is less effective in clearing senescent cells in older individuals. Determining whether the abundance/role of CD8+ NKG2C+ T cells is altered during acute and/or chronic injury would therefore be of interest.

Another important component for tissue regeneration is the extracellular matrix (ECM) and its cross-talk with cells in the surrounding microenvironment. Interestingly, the soluble SASP is known to induce ECM remodelling and stiffening, which has previously been shown to alter immune cell recruitment during ageing [136]. In fact, alterations to the ECM as a result of changes in matrix stiffness impair the access of immune cells to senescence-enriched tissues [136]. Furthermore, stromal cells such as fibroblasts are responsible for regulating tissue structure through deposition of the ECM, as well as supporting homeostasis through the secretion of cytokines, chemokines, growth factors and other key signalling proteins [136] (figure 4). We previously discussed the importance of the SASP in the release of soluble factors which influence the surrounding tissue microenvironment and maintain tissue homeostasis. It has been reported that extensive cross-talk exists between senescence-associated stromal populations that are known to accumulate during ageing and an immunosuppressive phenotype.

### 4.4. The parallels between macrophages and senescent cells

As well as the well-documented phagocytic role of macrophages, it was recently shown, for the first time, that chemotherapy-induced senescent cells (CISCs) are capable of engulfing both neighbouring senescent cells or non-senescent tumour cells in a macrophage-like fashion. It is important to note is that this behaviour was noted after chemotherapy treatment. However, this behaviour is also triggered by the administration of nutlin, which activates p53 without causing genotoxic stress [141]. This fascinating discovery that senescent cells, in the correct environment, can acquire a phagocytic phenotype, is suggestive of a potential survival advantage.

This newly found ability of senescent cells brings to light parallels with macrophages, whereby both macrophages and senescent cells secrete factors that elicit matrix remodelling and immunomodulation [142,143], and express metabolic markers, such as CD38 [144]. Indeed, cannibalism by breast cancer cells has been suggested to play a role in the induction of senescence [141]; thus, it will be important to investigate the role of cannibalism of senescent cells in a range of different contexts, including development, ageing and regeneration/repair. In particular, it will be interesting to determine the role of such cells in senescent cell accumulation, and whether cannibalism may play a role in cell clearance following injury. Furthermore, it will be pivotal to determine what cell types these cannibalistic cells preferentially engulf, and whether there are certain characteristics which lead to their engulfment, in order to determine whether, if such cells exist outside the context of cancer, they are beneficial or detrimental to the process of regeneration and repair.

### 4.5. The ageing immune system and immunosenescence

As the body ages, the innate immune system gradually declines, a phenomenon now termed immunosenescence [145], resulting in decreased effector immune cell function; and healthy tissue renewal rate decreases dramatically [146]. As a result, the aged tissue microenvironment accumulates senescent cells, such as SASP-associated fibroblasts, and gains the infiltration of immune infiltrates such as immunosuppressive myeloid-derived suppressor cells (MDSCs) and T regulatory cells. MDSCs are a heterogeneous population of cells of myeloid origin which repress T cells via the secretion of arginase 1, TGF*β* and reactive oxygen species (ROS), while T regulatory cells have a role in regulating or suppressing other cells in the immune system (reviewed in [147]). It has been suggested that this alteration in tissue microenvironment may encourage the development of pathological conditions such as cancer, and allows for the expansion of cancer cells unabated by the immune system. Indeed, in the event of chronic injury, it is certainly possible that this leniency of the immune system, or impaired immune surveillance, may also have similar effects, for example, by allowing the accumulation of senescent cells. In addition, the immunosenescence of effector T cells, NK cells, macrophages and dendritic cells is known to lead to a dramatic decrease in their cytotoxic activities and infiltration within an aged tumour-promoting microenvironment [148]. This has been supported by the observation that there are systemic increases in immunosuppressive M2 macrophages and N2 neutrophils in elderly people (reviewed in [149]), which may contribute towards an immunosuppressive phenotype. This has been shown in a model of senescence induction, using the fibroblasts accelerate stromal-supported tumorigenesis (FASST) mouse, where the authors observed an accumulation of senescent cells, as well as an increased number of MDSCs and regulatory T cells, which was not observed in a younger tissue microenvironment. Furthermore, this increase in the number of MDSCs and T reg cells in healthy mice was found adjacent to senescent cell populations and was found to primarily be due to the secretion of IL-6 [148]. Moreover, the deletion of perforin (*Prf1*), an essential component of immune surveillance of senescent cells, leads to the accumulation of senescent cells in the liver [150] and a general increase in the accumulation with advanced age, resulting in chronic inflammation, fibrosis and tissue damage [117]. Thus, this process of transient SASP and senescent cell clearance in young versus aged mice during the natural process of ageing shows very clear similarities with the occurrence of the senescence programme and the cycle of clearance/accumulation of senescent cells in acute and chronic injury, respectively.

A well-characterized mechanism by which senescent and apoptotic cells are regulated by macrophages is via their ‘eat me’ signals. However, senescent cells often express the ‘don't eat me’ signal CD47, a cell surface protein mediated by upregulation of NF*κ*B, which impairs phagocytosis by macrophages by binding to the inhibitory receptor signal regulatory protein alpha (SIRP*α*) found on the cell surface of macrophages. Conversely, senescent cells also appear to upregulate their expression of MHC class II molecules on their cell surface, which aid in their recognition by CD4+ T cells [123,132]. Furthermore, it has recently been identified that senescent cells also express an oxidized form of vimentin on their cell surface, which eventually gets released into the bloodstream, suggesting that oxidized proteins are capable of being recognized by the humoral innate immune system. This suggests that such proteins form part of a senescence-surveillance mechanism by acting as ‘eat me’ signals, and this process may become impaired with age [133]. Modified vimentin is already known to be expressed on the surface of immune cells including apoptotic neutrophils [151–153] and T cells [154], which eventually leads to elimination by macrophages [155]**.** In addition, it has been found that radiation-induced proteins found on the surface of apoptotic cells can interact with vimentin found on the surface of neighbouring phagocytes, leading to their removal by macrophages [155]. Understanding the mechanisms which regulate macrophage phagocytic ability and subsequent senescent cell clearance will be vitally important for developing future therapeutic approaches to aid regeneration and improve healthspan.

Senescent cells are also capable of communicating with neighbouring cells through the direct transfer of proteins. In 2015, it was shown by Biran *et al.* [130] that proteins were transferred to NK and T cells, and that this process was facilitated by immune surveillance of senescent cells by NK cells, resulting in their activation and increased cytotoxicity. The protein transfer was dependent strictly on cell–cell contact and CDC42-regulated actin polymerization, and partially mediated by cytoplasmic bridges. This raises the possibility that senescent cells may use intracellular protein transfer to induce senescence in neighbouring cells or perhaps even to promote survival. Furthermore, it is possible that this process may be important for regeneration, by allowing the cell–cell transfer of molecular mediators between senescent and regenerative or supportive cells. Indeed, the authors found that compared with normal cells, senescent cells preferentially use intercellular protein transfer (IPT) to regulate their own self-elimination by immune cells and communicate with epithelial cells. To support this, a variety of other studies have shown that IPT is used to both initiate and modulate immune responses which ultimately support cell survival [156–158]. Further investigation is required to comprehensively determine the transfer of material between macrophages and other immune cells during senescence and regeneration/repair. The recent publication of a proteomic database of soluble proteins and exosomal cargo SASP factors will undoubtedly lead to a more thorough understanding of cell communication and senescence [14].

While more is becoming known regarding the role of senescent cells and macrophages or the immune system in regeneration and repair, there remain many open questions. For example, it is still unclear whether macrophages themselves become senescent in the context of injury and whether this may contribute to the accumulation of senescent cells. Indeed, while damage to the immune system leads to the failure to clear senescent cells, contributing to their persistence in tissues, such immune failure may be in part due to immunosenescence, leading to immune cell dysfunction**.** Hall *et al.* [128] previously reported the observation of p16^INK4a^ and Sa-β-Gal expression in macrophages suggesting that senescent cells could spread senescence to immune cells. However, the authors later reported that the expression of these markers was not due to a spread of senescence, but rather acquired as part of a physiological response to immune stimuli [134]. This is consistent with reports that p16^INK4a^ is involved in macrophage polarization [135]. Thus, whether immune cells become senescent during injury or disease requires further investigation; however, it has been reported that immune cells undergo a progressive decline in function, known as immunosenescence, which has been reported to contribute to senescent cell accumulation [159,160]. Furthermore, if macrophages become senescent, do they still retain their ability to clear target cells, including other senescent cells? If this is the case, it is likely that macrophages contribute to the process of ‘inflammaging’, including immunosenescence, especially if senescent macrophages are found to promote a pro-inflammatory environment. Furthermore, understanding other mechanisms by which macrophages become damaged which cause changes in their function and or/phenotype during injury will also be important when considering therapeutic approaches.

### 4.6. Senescent cell accumulation and pathology

Other outstanding questions include whether there is a ‘tipping point’ at which senescent cells must accumulate to before the tissue becomes dysfunctional, and whether a similar process occurs at the point where macrophages are unable to clear senescent cells. As previously mentioned, the specific mechanisms by which macrophages interact with senescent cells remains to be elucidated. Understanding the molecular mechanisms which govern these interactions and which regulate macrophage function will be critical for the progression of therapies and the understanding of chronic diseases. In line with the interaction of senescent cells with CD4+ T cells to evade immune surveillance, it would be beneficial to further characterize whether similar mechanisms exist between macrophages and senescent cells. Finally, while various studies have individually indicated the role of different components of the senescence programme in the regenerative response, a comprehensive study linking together these processes remains to be undertaken. While the transient SASP is beneficial for regeneration and sustained SASP signalling promotes senescent cell accumulation, a comprehensive study of the ‘waves’ of the SASP in repair/regeneration, the point at which this process becomes detrimental, and the exact mechanisms which govern this response and how they interact with each other is still lacking.

## 5. Therapeutic manipulation of macrophages for tissue regeneration/repair

As covered in previous sections, the role of macrophages in maintaining tissue homeostasis, clearing senescent cells and promoting regeneration is beginning to be better established. Due to the innate role of macrophages in these processes, an emerging idea of treating diseases/age-related pathologies related to senescent cell accumulation falls within the realm of manipulating macrophage function. Such ideas include treating Alzheimer's disease [161], myocardial infarction [162], cancer [163] and inflammatory diseases [164] such as rheumatoid arthritis, with macrophage therapy.

### 5.1. Macrophage engineering approaches

One method of achieving a therapeutic approach is by engineering macrophages to ignore ‘don't eat me’ signals found on the surface of senescent cells. As previously mentioned, the CD47–SIRP*α* axis allows senescent cells to avoid removal by the immune system; therefore, blocking this axis may be an effective treatment against the accumulation of senescent cells in ageing and injury. Molecules which target this axis have already been developed and include those that target CD47, as well as SIRPα specifically, alongside bispecific targeting agents. The use of these agents has been well characterized in cancer, where anti-CD47 antibodies are currently in clinical trials [111]. Taking into account the role of macrophages in blocking the CD47–SIRPα axis, engineering macrophages to target CD47 may be effective. For example, similar to CAR-T cells, Chimeric Antigen Receptors for Phagocytosis (CAR-Ps) are designed to phagocytose specific targets, and it has been suggested that engineering macrophages to target CD47 may be an effective anti-tumour therapy [111]. Indeed, the use of these macrophages may prove effective in the clearance of chronic senescent cell accumulation following injury. However, there remain concerns over the manipulation of the CD47–SIRPα axis for therapeutic use; including, but not limited to, the consensus that the CD47–SIRPα interactions demonstrated in mouse studies may not completely or as efficiently translate to human [165]; the discovery that the clustering of CD47 can also influence the interaction between CD47 and SIRPα, and that the anti-CD47 non-blocking antibody 2D3 increases CD47 clustering [166,167], and the considerable functions of CD47 that are independent of SIRPα, which instead act upon SIRPγ or Thrombospondin 1 (TSP1), leading to potential off-target effects on T cells [168,169]. Another possibility is to increase the phagocytic capability of macrophages, by increasing ‘eat me’ signals. Defective expression of the ‘eat me’ signal calreticulin, a ligand required for activation of engulfment receptors on phagocytic cells, results in cellular resistance to efferocytosis, and apoptotic cells fail to be cleared by neighbouring macrophages [170]. A proportion of aged and cancerous cells are susceptible to being ‘labelled’ by macrophage-secreted calreticulin and are subsequently cleared from tissue [171]. Thus, the overexpression of calreticulin on senescent cells may provide a manner to increase phagocytosis and clearance from the tissue by macrophages. A third option involves the use of allogenic macrophages or administration of induced pluripotent stem (IPS) cell-derived macrophages from young donors [172]; however, whether young macrophages have better phagocytotic ability is not clear, with some studies reporting reduced ability with age [173], while others report no difference [174]. Moreover, differences appear to be related to the tissue from which they are derived and whether they are tissue resident or infiltrating [175]. Thus, macrophage targeting may be a therapeutic option for the clearance of senescent cells *in vivo*.

### 5.2. Senolytics

An alternative way to clear senescent cells is via the use of drugs that kill senescent cells, known as senolytics. It has been shown that the removal of senescent cells using senolytic drugs, such as ABT-263, which work by targeting proteins involved in the apoptosis pathway (such as BCL-xL and BCL-2), have been successful in removing senescent cells *in vitro* and *in vivo* [73,176]. In particular, ABT-263, which signals through the anti-apoptotic proteins BCL-xL and BCL-2, has proven effective in eliminating senescent cells in mouse models [73,177] and is currently undergoing phase 1 clinical trials in cancer patients [178]. Moreover, dasatinib plus quercetin (DQ), which not only targets BCL-2 family members, but also HIF-1α, PI3-kinase and p21-related anti-apoptotic pathways [179], has proved efficient at improving physical dysfunction in idiopathic pulmonary fibrosis (IPF) patients [180]. However, there remain limitations with the use of senolytics, largely due to lack of specificity, bioavailability and their route of administration. Indeed, if macrophages themselves are found to become senescent in the context of injury, it may be necessary to combine work on senolytics to include those that can target macrophages as well as other cells. Alternatively, a therapy that activates gene networks to promote a ‘younger’ phenotype in macrophages could provide a realistic option. This is especially important due to the critical role macrophages play in generating a regeneration-permissive environment needed for tissue repair.

As previously mentioned, while senolytics have proven effective in killing senescent cells, they retain broad-spectrum activity, especially considering the heterogeneous nature of the senescence programme. In 2020, Cai *et al.* [181] developed a new prodrug named SSK1 which is specifically activated by β-galactosidase (β-Gal) activity, believed to be a well-documented marker of senescent cells. The prodrug was shown to eliminate senescent cells, as well as re-establishing low-grade inflammation and restoring some measures of function. This novel drug therefore represents a more selective method of deleting senescent cells in a wide range of cell types and tissues [181]. In addition to reducing the number of senescent cells in general, the prodrug was found to decrease the number of SA-β-Gal positive macrophages in injured lungs and aged livers, which was accompanied by a reduction in inflammation-related cytokines. This suggests that the removal of macrophages themselves may be beneficial. Here it is important to note the previously mentioned study by Hall *et al.* in which the authors reported that senescent macrophages were evident in young mice, entirely due to their expression of the markers p16^INK4a^ and β-galactosidase [128]. However, the expression of these markers was later described to be markers of macrophage polarization and response [134]. Importantly, this highlights the need for better methods for the identification of senescence in immune cells. Moreover, since senescent cells do not have any one specific universal marker, and due to their dynamic nature, that their markers can alter over time, the specificity of any drug aimed at targeting senescent cells alone is problematic.

### 5.3. Genetic engineering approaches

As the removal of macrophages themselves tends to lead to severe consequences, an alternative approach is to alter their gene expression in order to induce certain phenotypes. This involves the expression of certain transcription factors, for example IRF5, which is involved in the polarization of macrophages towards an inflammatory phenotype, which in turn prevents healing and promotes inflammation. Thus, manipulating macrophages to express lower levels of IRF5 could be a promising strategy [182]. Furthermore, the delivery of microRNAs or other molecules through methods of delivery such as liposomes may be a promising method for genetic manipulation worthy of further investigation [183,184].

Regarding genetic manipulation, an interesting aspect of senescence that is yet to be thoroughly explored is the regulation of gene expression at the epigenetic level. An interesting idea in regenerative medicine is that pluripotency factors can be expressed in senescent cells, to promote re-entry into the cell cycle and modify gene expression profiles. In addition to this, there has been interest in the use of epigenetic factors to promote reprogramming, as senescent cells display a repressive chromatin configuration, which is thought to stably silence proliferation-promoting genes, while also activating the SASP. In particular, histone modifications such as H3K27me3 have been associated with upregulation of the SASP in senescent cells [185]. Interestingly, in a recent study, it was also shown that the activation of the senescence programme leads to the remodelling of the epigenetic landscape by recruiting BRD4, a transcriptional and epigenetic regulator, which activates newly activated super-enhancers located next to SASP genes [186]. In addition, BRD4 was found to be critical for the SASP and paracrine signalling, and in senescence immune surveillance. Importantly this study revealed how cells can activate immune-modulatory genes required for paracrine immune activation and a tumour-suppressive immune surveillance programme. This may indicate the way in which senescent cells are regulated during homeostasis and may provide targets for regeneration. Indeed, the inhibition of BRD4 disrupts the ability of immune cells to target and eliminate pre-malignant senescent cells *in vitro* and *in vivo* [186], suggesting the effectiveness of this approach.

### 5.4. Epigenetic targeting

To complement macrophage genetic engineering, targeting epigenetic enzymes acting on the chromatin in senescent cells may also be an effective approach to switch on gene regulatory networks associated with a ‘younger morphology’ and may contribute to regulating the SASP. This approach is promising, as through the identification of ‘master regulators’ it allows the regulation of a large number of intricately interlinked genes and gene networks, instead of the manipulation of small subsets. Indeed, senescent cells are known to remodel the epigenetic landscape in order to induce the expression of genes implicated in cellular defence and the inflammatory response, many of which are characterized as part of the SASP [17]. Furthermore, alterations in epigenetic regulation have been associated with immunosenescence, as Menin promotes histone acetylation at the *Bach2* locus, thereby suppressing T cell senescence, and subsequently, immunosenescence [187]. Indeed, this approach also relates to macrophages and may be successful in altering macrophage polarization.

### 5.5. Altering macrophage behaviour

Phagocytosis in macrophages is regulated through activation and inhibition of receptor signals. Activating receptors of macrophages sends a phagocytic signal that induces the ‘eat’ process. Importantly, when targeting senescent cells/macrophages for the purpose of regeneration/repair, it is important to note that an optimal result will probably be achieved through the careful, timely and balanced manipulation of this process. Indeed, the literature has pointed towards an important role of senescent cells in the initiation of the early stages of repair and of macrophages in clearing these cells in the first transient ‘wave’ of repair. It was not until recently that the soluble SASP was identified to have two distinct functional stages, the first stage being a highly anti-inflammatory stage enriched by TGFβ. Thus, the inhibition of this process as demonstrated by previous studies [188] is likely to have negative impacts on repair. However, the activation of macrophages at later points where senescent cells are found to accumulate is likely to be beneficial. Indeed, identifying the time points at which intervention is needed/most effective in different organ systems will be one of the most challenging aspects of developing effective therapies.

## 6. Conclusion, open questions and perspectives

From the initial thoughts that senescence was merely a natural result of ageing, our understanding of the role of senescence in ageing and human disease has greatly evolved. We are now aware that the senescence programme has more complex roles, notably in tissue regeneration and repair, beyond our current scope of knowledge. Furthermore, the crucial role of macrophages in repair and regeneration has also started to be demonstrated in the last decade, due to their well-reported role during injury. However, to date many questions remain regarding their specific role in the regulation of senescent cells following injury in different organ systems, which is complicated by the highly heterogeneous nature of the senescence programme, which directly influences macrophage function. Thus, to be able to target senescence for therapeutic means, a number of open questions need to be addressed, such as the following. (i) How do senescent cells interact with macrophages, and how does this change in different phases or ‘waves’ of the regenerative response? (ii) How do changes in the microenvironment trigger epigenetic/genomic/phenotypic changes in macrophages? (iii) At what point do senescent cells have to accumulate to before macrophages are unable to clear them, or when do senescent cells start to evade clearance by the immune system? Importantly, answering these questions will better elucidate how senescent cells and macrophages act in concert in the context of injury and repair, and will guide the development of treatments targeting senescent cells for therapeutic means.

## Data accessibility

This article has no additional data.
